# Changes in Left Ventricular Global and Regional Longitudinal Strain During Right Ventricular Pacing

**DOI:** 10.14740/cr454w

**Published:** 2016-02-20

**Authors:** Alaa Solaiman Algazzar, Azza Ali Katta, Khaled Sayed Ahmed, Nasima Mohamed Elkenany, Maher Abdelaleem Ibrahim

**Affiliations:** a Cardiology Department, National Heart Institute, Cairo, Egypt; b Cardiology Department, Faculty of Medicine, Al-Azhar University, Cairo, Egypt

**Keywords:** RV pacing, Longitudinal strain, Relative strain

## Abstract

**Background:**

Our study aimed to demonstrate the short-term impacts of right ventricular apical pacing (RVAP) and right ventricular septal pacing (RVSP) on left ventricular (LV) regional longitudinal strain (RLS) and global longitudinal strain (GLS) in patients with preserved ejection fraction (EF). LV strain and functions may be altered by RVAP. RVSP might be a better alternative. The detrimental effect of right ventricular (RV) pacing may be mediated by regional LV impairment.

**Methods:**

Sixty-two patients indicated for permanent pacemaker implantation and preserved LV systolic function were included. Dual chamber pacemakers were implanted in all patients. Patients were divided into two groups according to RV lead position: group A (RVAP, n = 32) and group B (RVSP, n = 30). Patients were examined at baseline and after 6 months of implantation for LV systolic functions, global and regional strain by echocardiography and 2D speckle tracking echocardiography.

**Results:**

Paced QRS duration was significantly shorter in group B compared to group A patients (P = 0.02). Regarding ventricular strain, there was no statistically significant difference between both groups at baseline measurements in comparisons of GLS, relative apical longitudinal strain (rALS) and RLS (P > 0.05). In contrast, there was statistically significant difference between both groups in results of GLS (P = 0.01) at 6 months. In addition, RLSs in septal, apical and rALS were affected after 6 months with P values of 0.02, 0.03 and 0.03, respectively.

**Conclusion:**

RVAP appears to worsen GLS more than RVSP, and the resultant decrease in apical strain is most correlated region to decrease in GLS.

## Introduction

Previous studies have demonstrated that atrioventricular (AV) sequential pacing has better hemodynamic effects over single ventricular pacing. A properly timed atrial systole improves stroke volume through the Frank-Starling mechanism. Higher left ventricular (LV) end-diastolic pressures and volumes, higher systolic and mean blood pressures and lower right atrial and pulmonary capillary wedge pressures have been reported with AV synchronous pacing [1].

The cardiac pacing at any point of the ventricle alters the natural heart activation and contraction pattern, as the stimulus conduction velocity is slower across the ventricular myocardium, when compared to that resulting from the specialized His-Purkinje system [2, 3]. In a review of 14 randomized studies, Shimony et al found that right ventricular mid-septal pacing (RVMSP) is associated with a better left ventricular ejection fraction (LVEF) during follow-up, compared with right ventricular apical pacing (RVAP) [4].

The physiological rationale behind pacing the septum rather than the apex is based on initiating the ventricular depolarization in the right ventricular (RV) septal wall, across the base of the mitral septal papillary muscle, where the first activation vector normally starts [5, 6].

Two-dimensional speckle tracking echocardiography (STE) allows detailed evaluation of left ventricular (LV) mechanics, including LV mechanical dyssynchrony, LV strain, and LV torsion [7, 8]. Myocardial strain may have the potential to identify reduced exercise capacity and poor prognosis at an early disease stage when traditional parameters fail [9, 10].

The purpose of our study was to compare short-term effects of RVAP and right ventricular septal pacing (RVSP) on LV global longitudinal strain (GLS) and regional longitudinal strain (RLS) in patients with preserved ejection fraction (EF).

## Patients and Methods

A total of 62 patients, who were indicated for elective permanent dual chamber pacemaker implantation according to current guidelines (class I), were included from May 2103 to August 2015. Adult patients with age less than 75 years with preserved EF were enrolled in the study after 6 months of implantation if they had more than 60% pacing dependence.

Patients were excluded if they had reversible causes for AV block; documented chronic heart dysrhythmias (slow AF); poor echo window; previous coronary artery disease detected by evidence of LV regional wall motion abnormalities at the echocardiogram or pathological Q waves in electrocardiogram, acute coronary syndrome and/or unstable angina; within 3 months of a myocardial infarction, coronary bypass surgery or a valve replacement, complex congenital heart disease, hypertrophic obstructive cardiomyopathy, severe mitral regurgitation, hemodynamically significant aortic stenosis, previous implanted pacemaker or ICD, post-AV junctional ablation; and terminal co-morbidities such as end-stage malignancy, end-stage renal or liver diseases.

After signing the written informed consent, patients were divided into two groups according to RV lead position: group A: RVAP (n = 32) and group B: RVSP (n = 30). Appropriate positioning of the electrode was confirmed fluoroscopically at the time of the pacemaker implantation, before the baseline visit. Documentation of lead position was acquired in each patient using three standard projections: anterior-posterior, 40° left anterior oblique (LAO 40°), and 40° right anterior oblique (RAO 40°) views referring to the method described by Mond et al [11]. The LAO fluoroscopic view appears to be the most desirable method to determine RV septal positioning [12, 13].

Patients were subjected to full history taking with history of the medications and complete general and local examination of the heart, chest and abdomen. The 12-lead ECG was done after programming pacemaker at heart rate of 90 beat/min (at time of doing ECG only) and duration of the QRS complex included the measurement of the time interval between the emission of the pacemaker spike and the end of the QRS complex (milliseconds (ms)).

Both groups were examined at baseline and after 6 months by conventional echocardiography and 2D speckle tracking. Calculations of morphometric parameters were done in accordance with the recommendations of American Society of Echocardiography [14]. The biplane Simpson’s rule was used for calculation of global LVEF. Pulsed tissue Doppler imaging (TDI) was used to obtain septal and lateral velocities for both E and S waves. Study participants underwent a transthoracic echocardiographic examination in the left lateral recumbent position using a commercial ultrasound scanner (Philips EPIQ 7, Philips healthcare, 3000 Minuteman Road, Andover, MA, USA) with an electronic ultrasonic transducer (X5-1).

Two-dimensional speckle tracking was performed to obtain longitudinal strain (LS) using apical four-, two-chamber, and long-axis views. Three consecutive cardiac cycles of each view were acquired during a breath hold at end-expiration. All the images were obtained at a frame rate of 50 frames to 80 frames per second. Timing of aortic valve closure was assessed looking at the aortic valve motion in the long-axis apical view and guided by ECG. All studies were digitally recorded and transferred to a dedicated workstation for further analysis.

Strain values from all segments were averaged to obtain a global longitudinal strain (GLS) value [15]. Also, the strain values of all segments at the three levels: basal, mid and apical segments of the LV were averaged to obtain three “regional” longitudinal strain (RLS) values. Strain values for each wall at all levels were obtained and averaged to obtain the average strain of the septal, lateral, inferior and anterior walls. The apex-to-base gradient in RLS was examined using absolute strain values as well as a relative apical LS calculated as [16]:

Relative apical LS = Average apical LS/(average basal LS + Average mid LS)

### Statistical analysis

The collected data were tabulated and statistically analyzed using SPSS version 22.0 for Windows (SPSS Inc., Chicago, IL, USA). Normal distribution of data was checked by Kolmogorov-Smirnov test. Categorical data were summarized as frequencies and percentages. Comparisons between the groups were performed using the unpaired Student’s *t*-test. Comparisons within the group were performed using the paired Student’s *t*-test. A probability value of 0.05 was considered statistically significant. Multiple linear regression analysis was performed to provide regression analysis and analysis of variance for one dependent variable as GLS levels at 6 months to QRS width, pacing percentage and regional strain parameters at baseline.

## Results

### Baseline characteristics

There is no significant difference between both groups regarding baseline characteristics and medications as outlined in Table 1.

**Table 1:** Baseline Characteristics of Studied Groups

Variables	Group A (n = 32)	Group B (n = 30)	P value
Age in years, mean ± SD	61.1 ± 7.5	58.3 ± 7.1	0.18
Body mass index, mean ± SD	26.59 ± 3.19	26.35 ± 3.78	0.78
Male, n (%)	6 (30)	19 (63.3)	0.31
Female, n (%)	14 (70)	11 (36.7)	
Smoking, n (%)	6 (30)	15 (50)	0.07
Diabetes, n (%)	4 (20)	5 (16.7)	0.67
Hypertension, n (%)	10 (50)	16 (53.3)	0.79
Dyslipidemia, n (%)	4 (12.5)	4 (13.3)	0.92
ACE inhibitors, n (%)	11 (34.3)	8 (26.6)	0.51
Beta blockers, n (%)	4 (12.5)	6 (20)	0.42
Calcium channel blockers, n (%)	4 (12.5)	1 (3)	0.18
Diuretics, n (%)	9 (28.1)	14 (46.6)	0.13
Insulin, n (%)	4 (12.5)	1 (3)	0.18
Metformin, n (%)	5 (15.6)	3 (10)	0.5
Gliclazide, n (%)	2 (6)	0	0.16
Glimpride, n (%)	1 (3.1)	4 (13.3)	0.14
Statin, n (%)	4 (12.5)	4 (13.3)	0.9

SD: standard deviation; ACE: angiotensin converting enzyme. % is the percentage within the group. n means number of patients within the group.

### Echocardiographic characteristics

In our study, there was no statistically significant difference in LVEF, septal and lateral E and S waves by pulsed tissue Doppler in both groups (P > 0.05) as shown in Table 2. After 6 months, the paced QRS width was significantly shorter in group B compared to group A patients (P = 0.02) as outlined in Table 2.

**Table 2:** Conventional Echocardiography Parameters, Pacing and QRS Duration at Baseline and After 6 Months Follow-Up for Studied Groups

	Group	Mean	SD	P value
Baseline				
Ejection fraction (%)	Group A	59.06	4.37	0.12
	Group B	61.40	4.88	
Septal E by TDI (cm/s)	Group A	10.90	1.55	0.64
	Group B	11.17	2.84	
Lateral E by TDI (cm/s)	Group A	12.71	1.67	0.09
	Group B	13.5	19.9	
After 6 months				
QRS duration (ms)	Group A	138.68	15.23	0.02*
	Group B	122.23	24.97	
Ejection fraction (%)	Group A	56.65	5.36	0.07
	Group B	60.36	6.21	
Septal E by TDI (cm/s)	Group A	10.78	1.56	0.75
	Group B	10.96	2.96	
Lateral E by TDI (cm/s)	Group A	11.92	1.90	0.12
	Group B	12.66	1.86	
Pacing percentage (%)	Group A	75.31	14.59	0.32
	Group B	72.03	10.96	

SD: standard deviation; TD: tissue Doppler imaging; cm: centimeter; ms: millisecond. % is the percentage within the group. *Significant P value.

### LV global and regional strains

**Figure 1:**
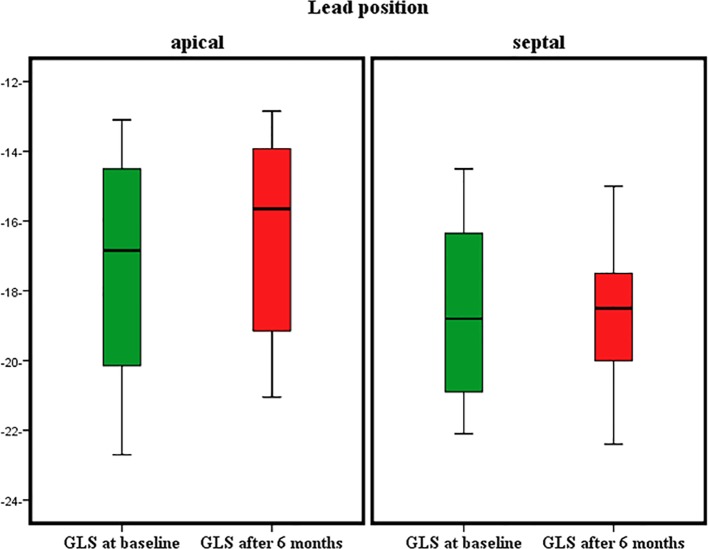
Global longitudinal strain (GLS) in both groups at baseline and after 6 months.

Regarding ventricular strain, our results showed no statistically significant difference between both groups at baseline measurements in comparisons of GLS, rALS and RLS (P > 0.05). In contrast, there was statistically significant difference between both groups in results of GLS (P = 0.01) at 6 months as shown in Table 3 and Figure 1.

**Table 3:** Comparison of Global and Regional Longitudinal Strain Between Studied Groups

	Group	Mean	SD	P value
Baseline				
Average septal LS	Group A	-16.48	5.43	0.41
	Group B	-16.18	3.93	
Average lateral LS	Group A	-17.66	5.33	0.77
	Group B	-18.01	4.19	
Average anterior LS	Group A	-16.83	5.06	0.18
	Group B	-18.52	4.82	
Average inferior LS	Group A	-18.70	3.98	0.52
	Group B	-19.29	3.24	
Apical LS	Group A	-17.94	4.29	0.08
	Group B	-16.92	4.31	
Mid LS	Group A	-15.98	5.44	0.16
	Group B	-17.83	4.93	
Basal LS	Group A	-20.05	3.44	0.44
	Group B	-20.71	3.25	
Relative apical LS	Group A	0.45	0.13	0.78
	Group B	0.51	0.11	
Global LS	Group A	-16.90	5.35	0.19
	Group B	-18.43	3.52	
After 6 month				
Average septal LS	Group A	-15.74	4.07	0.02*
	Group B	-18.37	2.97	
Average lateral LS	Group A	-15.68	4.99	0.9
	Group B	-15.59	3.02	
Average anterior LS	Group A	-15.28	5.40	0.1
	Group B	-17.56	5.38	
Average inferior LS	Group A	-18.57	3.68	0.5
	Group B	-19.28	3.24	
Apical LS	Group A	-15.32	3.74	0.03*
	Group B	-16.99	2.10	
Mid LS	Group A	-15.23	4.49	0.4
	Group B	-16.76	4.14	
Basal LS	Group A	-18.99	2.70	0.3
	Group B	-19.66	2.71	
Relative apical LS	Group A	0.39	0.11	0.03*
	Group B	0.50	0.12	
Global LS	Group A	-15.23	4.11	0.01*
	Group B	-18.29	2.09	

SD: standard deviation; LS: longitudinal strain. % is the percentage within the group. *Significant P value.

In addition, RLSs in septal, apical and rALS were affected after 6 months with P values of 0.02, 0.03 and 0.03, respectively as shown in Table 3.

### Correlation

**Figure 2:**
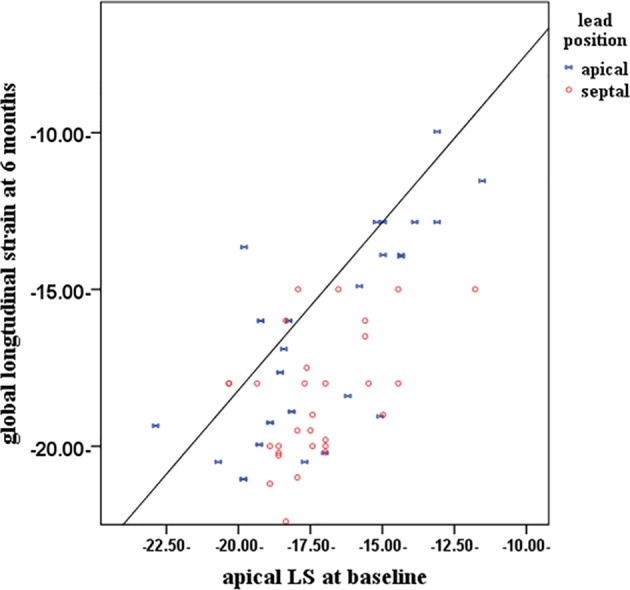
Scatter plot graph showing the relationship between global longitudinal strain (GLS) after 6 months and apical longitudinal strain at baseline (R = 0.622, P = 0.001). LS: longitudinal strain.

Figure 2 shows positive relationship between apical strain at baseline and GLS at 6 months with (R = 0.622, P = 0.001). Other regional strains failed to show any significant correlation with GLS at 6 months except QRS width (R = 0.352, P = 0.03) as shown in Table 4. On multivariable linear regression analysis, apical strains at baseline (b = 0.471, P < 0.001) and QRS duration (b = 0.257, P < 0.049) were independently related with GLS.

**Table 4:** Correlation Between GLS and Different Variables at Baseline

Variables controlled for lead position	GLS at 6 months	
	R	P value
QRS duration	0.352	0.03*
Apical LS at baseline	0.622	0.001*
Mid LS at baseline	0.233	0.07
Basal LS at baseline	0.189	0.29
Average anterior wall LS at baseline	0.218	0.09
Average inferior wall LS at baseline	0.209	0.1
Average lateral wall LS at baseline	0.216	0.09
Average septa wall LS at baseline	0.252	0.054

GLS: global longitudinal strain; LS: longitudinal strain. *Significant P value.

## Discussion

Two-dimensional STE is a feasible echocardiographic technique with an angle independency and high frame rate, which is capable of obtaining any directional strain. According to previous reports, the assessment of global systolic function by speckle tracking-based GLS was superior to the standard variables such as LVEF and wall motion score index for the prediction of outcome in patients undergoing echocardiography with good reproducibility [8, 17, 18]. We evaluated the LV GLS and RLS using a speckle tracking-based strain during RV pacing, addressing the hypothesis that GLS and RLS during systole was more deteriorated in RVAP than that in RVSP.

This study extends our understanding of how different RV pacing sites influence cardiac performance. The RV apex is the traditional site for ventricular pacing due to technical aspects such as the electrode design and the ease of the apical approach [19-21].

In our results, GLS was preserved in RVS lead than RVA lead position mostly as a result of Purkinje fiber recruitment. Impulse conduction using Purkinje fibers would promote depolarization in a more physiological fashion through the rapid conduction of these specialized muscle fibers. Furthermore, it is proposed that RVS lead pacing may assist inter- and intra-ventricular conduction by recruitment of circumferentially orientated myocardial fibers [22]. In support of these hypotheses, this study found that during pacing from the RV mid-septum, the QRS complexes became narrower, reflecting a reduction in electrical delay.

Meta-analysis by Shimony et al concluded that non-RVAP generally was not inferior to RVAP and that the longer the study period, at least more than 1 year, the more likely the result would favor non-RVAP. A review of individual studies shows that other sources of variation are also important, including duration of follow-up, lead position, percentage pacing, and baseline LV function [4].

In our study, we included only patients with preserved EF as baseline LV function is another potentially important determinant of the LV response to pacing. In a previous acute pacing study, RVAP in normal LV function produced little in the way of dyssynchrony but as baseline LV function worsened, the amount of dyssynchrony was greater. This would suggest that baseline LV function is important in the response to RV pacing [23].

In contrast to our results, the protect-pace study showed that pacing from either RVA lead or RVS lead position results in a small but statistically significant reduction in overall LV function over a 2-year period, but RVSP does not confer any protective (or detrimental) effect on LV systolic function over RVAP. Thus, there is no current indication to change standard pacing practice with regard to RV lead placement, as this remains a safe and effective treatment for a high-degree AV block [24]. But the primary endpoint of this study is the change in EF and secondary end-points were death and hospitalization for heart failure, atrial fibrillation (AF) burden, changes in brain natriuretic peptide (BNP) levels, and 6-min walk test. They did not use any quantification methods as strain or dyssynchrony. The EF, derived only from changes to the ventricular lumen, does not necessarily reflect myocardial muscle or sarcomeric shortening. Even if contractility is reduced, compensatory mechanisms (i.e., ventricular dilatation, geometry changes) can still assure that stroke volume remains normal at least at rest [25]. Previous studies have suggested that changes in LV function might occur after 12 - 18 months [12, 26, 27].

We found that RVAP worsens apical, relative apical and septal regional strain more than RVSP. In our study, the resultant decrease in apical strain is correlated with GLS. This was the same as Makoto et al, who found that septal apical strain was significantly lower in RVA than that in non-RVA at baseline and 2 years. Overall, change in GLS for 2 years was significantly correlated with septal apical strain at 2 years [28]. RVAP leads to increasing intra-ventricular dyssynchrony, even though mid-septum lead position was believed to normalize LV dyssynchronization caused by RV pacing [29].

This phenomenon can be explained by altered cardiac mechanics due to lead position. Myocardial fiber orientation in the LV wall is helix like and LV fibers are in two opposite directions in subepicardium and subendocardium [30-33]. Contraction of these obliquely oriented fibers creates a twisting motion of the LV. Torsional deformation is determined by the net effect of positive torsional deformation forces developing in the subepicardium and negative torsional deformation forces generated in the subendocardial fibers. The loss of torsion results from the reduction of counterclockwise apical rotation. These severe impairments of apical strain can not generate sufficient apical rotational movement, which is the main determinant of LV systolic twist [34, 35].

It is important to note that measures of regional or segmental function such as myocardial strain may actually reflect “global” systolic function better than the EF. EF can be determined by both myocardial strain and end-diastolic wall thickness [36]. Several reports have confirmed a significant correlation between LV torsion, apical rotation, strain variables and EF. Apical rotation was demonstrated to be an independent predictor of EF reduction [34-38]. In two different studies, healthy subjects were evaluated in sinus rhythm and later with AV synchronous RVAP. Acute RVAP causes reductions in LV longitudinal strain and twist. So this should be the next step in our analysis [39, 40].

From the above mentioned studies, we can infer why affection of apical strain is strongly correlated to GLS, and late on to affect EF. The recognition of abnormal LV strain patterns may provide longitudinal clues to LV dysfunction in chronically paced patients and potential novel indices of effective CRT interventions to reverse these abnormalities.

### Limitations

We could not evaluate other speckle tracking-derived parameters in a circumferential and radial direction. The present analysis focuses on the short-term impact of RVAP on LV functions and does not provide information about the long-term consequences. However, previous studies have demonstrated acute effect of RVAP on GLS [39, 40]. We considered GLS as a surrogate to EF. However, we should evaluate the effect of lead position on apical rotation and LV torsion. We could not fully achieve the blinding of the echocardiographer because the additional lead was visible during examination. Finally, we need a large trial that unifies clinical and quantification methods of follow-up including understanding cardiac mechanics to evaluate effect of RV lead position.

### Conclusion

RVAP appears to worsen GLS more than RVSP, and the resultant decrease in apical strain is most correlated region to decrease in GLS.
